# Academic performance, educational aspiration and birth outcomes among adolescent mothers: a national longitudinal study

**DOI:** 10.1186/1471-2393-14-3

**Published:** 2014-01-15

**Authors:** Yiqiong Xie, Emily Wheeler Harville, Aubrey Spriggs Madkour

**Affiliations:** 1 Department of Epidemiology, Tulane University School of Public Health and Tropical Medicine, 1440 Canal St. SL-18, New Orleans, LA, 70114, USA; 2 Department of Global Community Health and Behavioral Sciences, Tulane University School of Public Health and Tropical Medicine, New Orleans, USA

**Keywords:** Adolescents, Birthweight, Gestational age, Academic performance, Educational aspiration

## Abstract

**Background:**

Maternal educational attainment has been associated with birth outcomes among adult mothers. However, limited research explores whether academic performance and educational aspiration influence birth outcomes among adolescent mothers.

**Methods:**

Data from Waves I and IV of the National Longitudinal Study of Adolescent Health (Add Health) were used. Adolescent girls whose first pregnancy occurred after Wave I, during their adolescence, and ended with a singleton live birth were included. Adolescents’ grade point average (GPA), experience of ever skipping a grade and ever repeating a grade, and their aspiration to attend college were examined as predictors of birth outcomes (birthweight and gestational age; n = 763). Univariate statistics, bivariate analyses and multivariable models were run stratified on race using survey procedures.

**Results:**

Among Black adolescents, those who ever skipped a grade had higher offspring’s birthweight. Among non-Black adolescents, ever skipping a grade and higher educational aspiration were associated with higher offspring’s birthweight; ever skipping a grade was also associated with higher gestational age. GPA was not statistically significantly associated with either birth outcome. The addition of smoking during pregnancy and prenatal care visit into the multivariable models did not change these associations.

**Conclusions:**

Some indicators of higher academic performance and aspiration are associated with better birth outcomes among adolescents. Investing in improving educational opportunities may improve birth outcomes among teenage mothers.

## Background

In 2011, there were 333,771 live births to mothers age 10-19 in the U.S [1]. Negative pregnancy and birth outcomes, such as infant mortality [2-4], stillbirth [5], congenital anomalies [6], preterm birth (PTB), and low birthweight (LBW) [7] disproportionately affect adolescent compared to adult mothers. Unfortunately, most research on determinants of birth outcomes has focused on adult rather than teen mothers. Given the large number of adolescent births and the related pregnancy complications, it is important to explore factors that influence birth outcomes specifically within the teenage group.

Among adult mothers, maternal educational attainment is positively linked with birth outcomes [8-14]. Lower education has been associated with elevated risks of low birthweight [11,13], preterm birth [8,11,14], small for gestational age [8,11,12], still birth [8], and infant death [8]. One study found that there was a positive linear relationship between maternal year of schooling and birthweight in black women, and a non-linear relationship in white women, with the optimal level of schooling with regard to birthweight being about 16 years and lower or higher levels of education associated with lower birthweight [9].

Although evidence of the association between maternal educational attainment and birth outcomes has been consistent, there remain uncertainties about the explanation for the association [9]. Fuchs argues that educated people invest in their own and their offspring’s health in terms of healthy behaviors and utilization of health care services [15]. Women with lower educational attainment are more likely to smoke during pregnancy [16-18], and some researchers reported that the relationship between lower maternal education and adverse perinatal outcomes was (partly) explained by smoking during pregnancy [11,14,19]. Other studies found that maternal education and smoking during pregnancy had independent effects on small-for-gestational-age births and child’s antisocial behaviors [12,16]. Women with lower education are also more likely to have less or delayed prenatal care [20-22].

The Healthy People 2020 agenda includes goals related to improving adolescents’ academic success and achievement [23]. Academic performance and educational aspiration during adolescence are found to be related to educational attainment in adulthood [24,25]. Rumberger reported that grade retention and poor academic performance were important factors that were predictive of dropping out of middle school [24]. Grade skipping, which is one form of grade acceleration [26], has been found to be associated with better future educational and occupational outcomes in science, technology, engineering, and mathematics among males, and with a higher likelihood of pursuing law degrees and medical degrees in females [27]. Academic performance and educational expectation have also been negatively associated with a number of risky behaviors, such as cigarette smoking [28-31], alcohol drinking [30,31], use of marijuana [30,31], early sexual initiation [32] and violence [33,34]. Thus it is reasonable to hypothesize that adolescents’ academic performance and educational aspiration might be related with birth outcomes through behavioral intermediaries.

However, while there has been growing literature on maternal educational attainment and birth outcomes among adult mothers, less research has been conducted exploring whether academic performance and educational aspiration influence birth outcomes among adolescent mothers. Teen mothers have been found to have poorer educational performance compared to other teens [35,36]. This raises the question of a potential relationship between adolescents’ academic performance, educational aspiration and birth outcomes.

The purpose of the present study is to examine how maternal academic performance and educational aspiration during adolescence are related to birth outcomes among adolescent mothers. In addition, we examine whether these relationships, if any, can be explained by maternal smoking during pregnancy and utilization of prenatal care. We use data from a nationally-representative survey of school-attending youth who have completed their teenage years and thus have complete data on teenage pregnancies. Results will add to existing literature on the determinants of birth outcomes among adolescents.

## Methods

### Data

Data from waves I and IV of the National Longitudinal Study of Adolescent Health (Add Health) contractual dataset were utilized [37]. Add Health is a prospective cohort study of a nationally-representative sample of youth enrolled in grades 7-12 in the 1994-95 school year (Wave I) [38]. Follow-up interviews were conducted in 1996 (Wave II), 2001 (Wave III), and 2007-08 (Wave IV). A multistage probability clustered sampling design was used to obtain its Wave I sample. The first stage was a stratified, random sample of all public and private high schools in the U.S. A feeder school was also recruited from each participating community. In-school surveys were attempted with all students attending participating schools; a total of 90,118 were completed. In the second Wave I sampling stage, a sample of adolescents was drawn for in-depth in-home interviews, consisting of a random core sample plus selected special oversamples; a total of 20,745 interviews were conducted at this stage. At Wave II, most students (except Wave I seniors) were eligible for re-interview; at Waves III and IV, all respondents to the Wave I in-home interview were eligible for re-interview. A total of 15,701 interviews were conducted at Wave IV (80.3% response rate). Sampling weights adjusted for both unequal probabilities of selection into the original sample and for loss to follow-up. The present study was deemed exempt by the IRB at Tulane University.

We applied a number of sample inclusion criteria. First, we limited to females who participated in Wave IV, as that was the Wave when all respondents had completed their teenage years and had complete teen pregnancy data. Second, we limited to female participants with valid sampling weights in order to make generalizations to the U.S. population. Third, we limited to females whose first pregnancies occurred during adolescence, after Wave I and ended with a singleton live birth to ensure the temporal ordering of predictors and outcomes (n = 978). Finally, we limited to females with complete information on all covariates. This left us with an analysis sample of 763 teen singleton live births.

### Measures

#### Outcomes

At Wave IV girls were asked about previous pregnancies and their outcomes. If they indicated they had given birth, they were asked “How much did the baby weigh at birth?”, “Was [baby’s name] born before or after [his/her] due date?” and then “How many weeks or days early/late was [baby’s name] born?” This was subtracted from 40 weeks to calculate gestational age. Birthweight was examined in kilograms and gestational age was examined in weeks.

### Predictors

#### Academic performance

We measured adolescents’ *grade point average (GPA)* based on their self-reported school performance in four subjects in Wave I in-home interview: English or language arts, mathematics, history or social studies, and science. Letter grades of A, B, C, and D were coded as 4, 3, 2, and 1, respectively and were averaged and dichotomized at the median (median = 2.5). In Wave I in-home interview adolescents were also asked whether they had ever skipped a grade [yes/no] and repeated a grade [yes/no].

#### Educational aspiration

Adolescents’ educational aspiration was measured from three questions in Wave I in-home interview asking how much they wanted to go to college, how likely they were to go to college, and how disappointed the adolescent thought his/her mother would be if the adolescent did not graduate from college. Responses were provided in a 5-point Likert scale, with higher scores indicating higher expectation or likelihood of going to college. Because of the likely high correlation between these items, we conducted a polychoric principal factor analysis with varimax rotation among the three questions, and the results suggested a single-factor solution (factor loading: 0.74, 0.77, and 0.71, respectively). We then created the *educational aspiration* scale by summing the items weighted by their factor loadings, with higher score indicating higher aspiration for college education. In bivariate and multivariable analyses, we standardized the score so that the regression coefficients represent change in outcome with a one-standard deviation change in the educational aspiration score.

We examined correlation among all predictor variables (i.e., GPA, grade skipping, grade repeating, and educational aspiration) and found low correlations, with polychoric correlation coefficients ranging from 0.01 to 0.34.

### Mediators

Based on prior literature, we included measures of *smoking during pregnancy* and *timing of prenatal care visit* as potential mediators of maternal education variables and birth outcomes. *Smoking during pregnancy* was assessed at Wave IV for each pregnancy reported. Respondents were asked to report on an ordinal scale how many cigarettes they smoked during their pregnancy (none/a few cigarettes but not every week/a few cigarettes a week but not every day/10 or fewer a day/11-20 a day/21-30 a day/31 or more a day). We dichotomized responses due to sparseness across smoker frequencies in the sample. The *timing of prenatal care initiation* was based on two questions asked about each pregnancy reported at Wave IV: “During this pregnancy with [partner] did you ever visit a doctor, nurse-midwife or other health care provider for prenatal care, that is, for one or more pregnancy check-ups?” and “How many weeks pregnant were you at the time of your first prenatal care visit?” Responses to these two questions were combined and categorized into three groups: initiated in the first trimester; initiated in the second trimester; and initiated in the third trimester or no prenatal care.

### Controls

We drew upon our previous analysis of predictors of birth outcomes in this cohort to determine confounders [39]. This analysis indicated effects of race, age at pregnancy, age at Wave I, body mass index (BMI) categories and parental education. Parental education was measured as the higher of either co-residential mother or father: less than high school diploma, high school diploma/GED or higher. BMI was categorized into four groups: underweight (BMI < 18.5 kg/m^2^), normal weight (18.5 kg/m^2^ ≤ BMI < 25.0 kg/m^2^), overweight (25.0 kg/m^2^ ≤ BMI < 30.0 kg/m^2^), and obese (BMI ≥ 30.0 kg/m^2^) [40].

### Analyses

All analyses were conducted in SAS (SAS Institute, Cary, North Carolina) using survey procedures, which apply population weights and adjust standard errors for non-independence between respondents due to school-based sampling. We began with descriptive statistics (means and percentages) for all analysis variables, then conducted bivariate analyses (t-test, ANOVA or linear regression) to test the crude relationships between analysis variables and birth outcomes. Finally, we implemented multivariable linear regression models to examine the relationship between maternal academic performance and educational aspiration and birth outcomes after controlling for other individual and family characteristics. For each birth outcome (birthweight, gestational age), we examined two models: one without potential mediator variables and the other with potential mediators. Mediation was assessed based on change in effect estimates after inclusion of the mediating variables [41]. Analyses were run stratified on race (Black / non-Black) given prior literature suggesting different associations between maternal education and birth outcomes according to race [9]. Significance testing was conducted at α = 0.05.

## Results

The characteristics of the study population are presented in Table 1. Average age at Wave I, when girls’ academic performance and educational aspiration were examined, was about 15 for both Black and non-Black girls. This was on average about two years before they gave birth. Black adolescent mothers had lower birth weight and a smaller proportion with GPA above median compared with non-Black mothers. The educational aspiration was higher in Black adolescents than in non-Black adolescents. A larger proportion of non-Black girls ever smoked during pregnancy than that in Black girls.

**Table 1:** Characteristics of teen mothers whose first pregnancy occurred during adolescence and after wave 1 at the add health study, 1996-2007 (N = 763)

	**Overall sample (n = 763)**	**Black (n = 244)**	**Non-black (n = 519)**	**P-value**
Birthweight (kg) (mean[se])	3.23(0.03)	3.10(0.05)	3.27(0.03)	<0.01
Gestational age (week) (mean[se])	39.29(0.11)	39.16(0.2)	39.33(0.12)	0.49
Baseline age (year) (mean[se])	15.33(0.14)	15.36(0.26)	15.32(0.15)	0.89
Age at pregnancy (year) (mean[se])	17.80(0.09)	17.67(0.13)	17.83(0.09)	0.20
Baseline BMI category (n [%])				0.03
Underweight	96(15.02)	25(10.88)	71(16.28)	
Normal weight	508(67.24)	155(63.43)	353(68.4)	
Over weight	119(13.07)	50(20.69)	69(10.74)	
Obese	40(4.68)	14(5.00)	26(4.58)	
Parental education ([n%])				0.41
≥HS	606(80.64)	213(83.44)	393(79.79)	
Less than HS	157(19.36)	31(16.56)	126(20.21)	
Educational aspiration(non-standardized) (mean[se])^*^	−0.09(0.06)	0.06(0.09)	−0.14(0.06)	0.05
GPA				0.04
Below average	312(42.99)	98(50.84)	214(40.59)	
Above average	451(57.01)	146(49.16)	305(59.41)	
Ever skipped a grade (n [%])				0.85
No	744(97.57)	239(97.78)	505(97.51)	
Yes	19(2.43)	5(2.22)	14(2.49)	
Ever repeated a grade (n [%])				0.20
No	618(79.82)	187(75.52)	431(81.14)	
Yes	145(20.18)	57(24.48)	88(18.86)	
Smoking during pregnancy (n [%])				<.0001
No	607(74.93)	230(93.81)	377(69.16)	
Yes	156(25.07)	14(6.19)	142(30.84)	
Initiation of prenatal care (n [%])				0.58
1^st^ trimester	541(69.14)	174(71.63)	367(68.37)	
2^nd^ trimester	154(21.77)	50(21.65)	104(21.81)	
3^rd^ trimester or no prenatal care	68(9.09)	20(6.71)	48(9.82)	

*Range of Educational aspiration score (non-standardized):−3.46, 1.23.

In bivariate analysis, none of the education variables was significantly associated with offspring’s birthweight among Black adolescents while higher educational aspiration and ever skipping a grade were related with higher birthweight in non-Black adolescents. Smoking during pregnancy was positively related with birth weight among Black adolescents (Table 2). Ever skipping a grade was also positively associated with offspring’s gestational age in non-Black girls. Smoking during pregnancy was associated with higher gestational age among Black adolescents (Table 3).

**Table 2:** Bivariate analysis between maternal characteristics, education variables and birthweight (kg) (N = 763)*

	**Black (n = 244)**		**Non-black (n = 519)**		**P-value for interaction**
	**Β(se)**	**P**	**Β(se)**	**P**	
Baseline age	−0.03(0.03)	0.41	0.004(0.02)	0.84	0.42
Age at pregnancy	−0.01(0.03)	0.82	−0.02(0.03)	0.59	0.84
Baseline BMI category		0.44		0.15	0.08
Underweight	0.13(0.16)		−0.16(0.07)		
Normal weight	ref		ref		
Over weight	0.17(0.12)		−0.10(0.14)		
Obese	−0.15(0.34)		0.04(0.10)		
Parental education		0.06		0.51	0.11
≥HS	ref		ref		
Less than HS	0.16(0.08)		−0.07(0.10)		
Educational aspiration standardized	−0.04(0.06)	0.46	0.07(0.03)	0.03	0.10
GPA		0.86		0.63	0.95
Below average	0.02(0.14)		0.03(0.07)		
Above average	ref		ref		
Ever skipped a grade		0.35		<0.01	0.09
No	ref		ref		
Yes	0.12(0.13)		0.48(0.15)		
Ever repeated a grade		0.44		0.21	0.63
No	ref		ref		
Yes	−0.05(0.07)		−0.11(0.08)		
Smoking during pregnancy		0.05		0.36	0.03
No	ref		ref		
Yes	0.24(0.12)		−0.06(0.07)		
Initiation of prenatal care		0.47		0.71	0.71
1^st^ trimester	ref		ref		
2^nd^ trimester	0.03(0.10)		0.05(0.07)		
3^rd^ trimester or no prenatal care	−0.11(0.09)		−0.001(0.12)		

*Figures presented are regression coefficients with standard errors.

**Table 3:** Bivariate analysis between maternal characteristics, education variables and gestational age (week) (N = 763)*

	**Black (n = 244)**		**Non-black (n = 519)**		**P-value for interaction**
	**Β(se)**	**P**	**Β(se)**	**P**	
Baseline age	0.18(0.10)	0.08	0.01(0.08)	0.87	0.20
Age at pregnancy	−0.05(0.10)	0.60	−0.07(0.09)	0.43	0.86
Baseline BMI category		0.68		0.47	0.94
Underweight	0.31(0.36)		−0.09(0.27)		
Normal weight	ref		ref		
Over weight	0.18(0.43)		−0.03(0.46)		
Obese	−1.60(2.10)		0.54(0.37)		
Parental education		0.16		0.27	0.08
≥HS	ref		ref		
Less than HS	0.54(0.38)		−0.35(0.32)		
Educational aspiration standardized	−0.07(0.19)	0.71	0.09(0.11)	0.39	0.44
GPA		0.54		0.62	0.42
Below average	−0.25(0.41)		0.14(0.28)		
Above average	ref		ref		
Ever skipped a grade		0.95		<0.01	0.29
No	ref		ref		
Yes	−0.06(0.88)		0.95(0.32)		
Ever repeated a grade		0.29		0.38	0.18
No	ref		ref		
Yes	0.36(0.33)		−0.28(0.32)		
Smoking during pregnancy		0.01		0.53	0.08
No	ref		ref		
Yes	0.92(0.36)		0.14(0.21)		
Initiation of prenatal care		0.16		0.10	0.95
1^st^ trimester	ref		ref		
2^nd^ trimester	0.66(0.38)		0.55(0.25)		
3^rd^ trimester or no prenatal care	−0.01(0.61)		0.28(0.46)		

*Figures presented are regression coefficients with standard errors.

Multivariable analysis of birthweight is presented in Table 4. Among Black adolescents, ever skipping a grade was associated with 200 grams increase in their offspring’s birthweight when controlling for other variables (p = 0.04). Among non-Black adolescents, ever skipping a grade and higher educational aspiration were associated with higher birthweight (p < 0.01 and p = 0.03, respectively). After adding the two potential mediators (i.e., smoking during pregnancy and prenatal care visit), the associations between the main predictors and birthweight remained largely the same, suggesting no mediation (model 2, Table 4). However, smoking during pregnancy was significantly positively associated with birthweight in Black girls.

**Table 4:** **Multivariable analysis between maternal characteristics, educational variables and birthweight (kg) (N = 763)**^**†**^

	**Black (n = 244)**		**Non-black (n = 519)**	
	**Model 1**	**Model 2**	**Model 1**	**Model 2**
Baseline age	−0.03(0.03)	−0.03(0.03)	0.004(0.02)	0.002(0.02)
Age at pregnancy	−0.004(0.03)	−0.001(0.03)	−0.03(0.03)	−0.02(0.03)
Baseline BMI category				
Underweight	0.17(0.16)	0.14(0.16)	−0.15(0.07)	−0.15(0.07)
Normal weight	ref	ref	ref	ref
Over weight	0.15(0.13)	0.15(0.13)	−0.11(0.13)	−0.12(0.13)
Obese	−0.17(0.35)	−0.21(0.37)	0.06(0.11)	0.05(0.11)
Parental education				
≥HS	ref	ref	ref	ref
Less than HS	0.13(0.08)^§^	0.15(0.08)^§^	−0.07(0.09)^§^	−0.07(0.10)^§^
GPA				
Below average	−0.002(0.14)	−0.01(0.14)	0.11(0.07)	0.12(0.07)
Above average	ref	ref	ref	ref
Ever skipped a grade				
No	ref	ref	ref	ref
Yes	0.20(0.10)^*^	0.22(0.09)^*^	0.50(0.17)^**^	0.49(0.17)^**^
Ever repeated a grade				
No	ref	ref	ref	ref
Yes	−0.09(0.07)	−0.11(0.08)	−0.08(0.08)	−0.08(0.08)
Educational aspiration	−0.05(0.05)^§^	−0.05(0.05)^§^	0.08(0.04)^*§^	0.08(0.04)^*§^
Smoking during pregnancy				
No		ref		ref
Yes		0.31(0.13)^*§^		−0.03(0.07)^§^
Initiation of prenatal care				
1^st^ trimester		ref		ref
2^nd^ trimester		0.001(0.09)		0.07(0.06)
3^rd^ trimester or no prenatal care		−0.06(0.08)		−0.01(0.13)

^*^P < 0.05; ^**^P < 0.01; ^§^P for interaction <0.05.

^†^Figures presented are regression coefficients with standard errors.

For gestational age (Table 5), none of the education variables was significantly related to birth outcomes in Black adolescent girls. Among non-Black adolescents, those who ever skipped a grade had around one week increase in offspring’s gestational age (p = 0.03). The addition of the two potential mediators did not change the association between grade skipping and offspring’s gestational age. Similar to the bivariate analysis, smoking during pregnancy was associated with increased gestational age among Black adolescents.

**Table 5:** **Multivariable analysis between maternal characteristics, educational variables and gestational age (week) (N = 763)**^**†**^

	**Black (n = 244)**		**Non-black (n = 519)**	
	**Model 1**	**Model 2**	**Model 1**	**Model 2**
Baseline age	0.19(0.10)	0.17(0.10)	0.03(0.10)	0.01(0.10)
Age at pregnancy	−0.13(0.12)	−0.11(0.12)	−0.11(0.10)	−0.09(0.10)
Baseline BMI category				
Underweight	0.46(0.42)	0.36(0.41)	−0.05(0.29)	−0.08(0.30)
Normal weight	ref	ref	ref	ref
Over weight	0.06(0.44)	−0.01(0.46)	−0.01(0.44)	−0.12(0.47)
Obese	−1.46(2.09)	−1.57(2.13)	0.64(0.36)	0.62(0.37)
Parental education				
≥HS	ref	ref	ref	ref
Less than HS	0.60(0.40)^§^	0.64(0.45)^§^	−0.43(0.28)^§^	−0.40(0.28)^§^
GPA				
Below average	−0.33(0.48)	−0.31(0.46)	0.31(0.29)	0.35(0.30)
Above average	ref	ref	ref	ref
Ever skipped a grade				
No	ref	ref	ref	ref
Yes	−0.28(0.87)	−0.18(0.88)	0.97(0.43)^*^	1.11(0.43)^*^
Ever repeated a grade				
No	ref	ref	ref	ref
Yes	0.11(0.32)	0.01(0.34)	−0.31(0.34)	−0.33(0.35)
Educational aspiration	−0.09(0.22)	−0.08(0.22)	0.14(0.15)	0.14(0.15)
Smoking during pregnancy				
No		ref		ref
Yes		1.14(0.53)*		0.18(0.23)
Initiation of prenatal care				
1^st^ trimester		ref		ref
2^nd^ trimester		0.41(0.36)		0.57(0.25)
3^rd^ trimester or no prenatal care		−0.07(0.66)		0.30(0.43)

^*^P < 0.05; ^§^P for interaction <0.05.

^†^Figures presented are regression coefficients with standard errors.

### Post-hoc analysis

As our study sample is a selected one including females whose first pregnancies occurred during adolescence and ended with a singleton live birth, we were concerned about potential sample selection biases that might have influenced our findings. We did a post-hoc analysis examining whether maternal academic performance and educational aspiration during adolescence predicted the likelihood of having teen pregnancy. Among 6,497 adolescent girls with valid sampling weight and non-missing values on all covariates, 1,406 (21.64%) ever had teen pregnancy. Among Black girls (n = 1,491), below-average GPA was associated with a higher risk of teen pregnancy (Odds Ratio [OR] = 2.22, 95% Confidence Interval [CI]: 1.51-3.26). Among non-Black girls (n = 5,006), below-average GPA, ever skipping a grade, and ever repeating a grade were related with a higher likelihood of teen pregnancy (OR = 2.10, 95% CI: 1.71-2.57; OR = 2.02, 95% CI: 1.08-3.77; and OR = 1.63, 95% CI: 1.23-2.17, respectively); higher educational aspiration was negatively associated with the risk of teen pregnancy.

## Discussion

Educational goals and achievements are likely to be particularly important for adolescents, as school is their major social and occupational context [42]. However, most studies that linked maternal education with birth outcomes used maternal educational attainment in adulthood or at the child’s birth [8-12,14]. Few studies have explored whether academic performance and educational aspiration during adolescence are associated with birth outcomes among adolescent mothers. In this study, data were collected prospectively, which allowed us to assess academic performance and educational aspiration prior to the teen pregnancy.

Multivariable analysis indicated that higher aspiration for college education was related to higher birthweight among non-Black adolescents. Higher educational aspiration could indicate a greater future orientation and greater social support [43] which would be associated with healthier pregnancies through health-protective behaviors, such as good nutrition and physical activity [44]. On the other hand, girls who do not have high aspiration for their educational futures may be reflecting a family or community not generally supportive of the girl or her goals, which might translate to reduced social support during pregnancy [45]. It also might indicate their life circumstances, which could include poor health or a community that does not have many people going to college [46,47]. This may be part of a fatalistic attitude towards pregnancy and pregnancy outcomes, which might lead to poorer self-care.

We found that grade skipping was associated with higher birthweight and gestational age among non-Black girls, as well as higher birthweight among Black girls. Grade skipping is one way to accomplish the concept of appropriate developmental placement [27,48]. It allows intellectually precocious students to experience more developmentally appropriate content by skipping over what they have already known or can easily learn [49]. It has been suggested to have merit as the intellectually talented students potentially gain intellectual development, interpersonal maturity and time when they advance more quickly through the educational system [27]. It is possible that adolescents who were accelerated had greater social support [50] and personal resilience and thus have healthier lifestyles and behaviors during pregnancy which lead to better birth outcomes.

No statistically significant relationship was found between adolescents’ GPA and either of the birth outcomes. However, we observed that among non-Black adolescents, those whose GPA was below average had higher birthweight and gestational age than those with GPA above average and the magnitude of the coefficients was larger than that for grade skipping. It is possible that this finding is (partly) due to the imperfect measurement of adolescents’ GPA; adolescents may have mis-reported their letter grades in Wave I in-home interview. Nonetheless, this was an unexpected finding. We tested the interaction between GPA and grade skipping, as well as interaction between GPA and grade repeating, to determine whether a low GPA represented more challenging courses relative to age, but neither was statistically significant (p > 0.05).

Past studies suggested that the effect of maternal education on birth outcomes could be accounted for by the health-related risk behaviors, particularly maternal smoking and alcohol use during pregnancy and late entry into prenatal care [11,14,15,19]. However, in our study the addition of smoking during pregnancy and prenatal care visit did not change the regression coefficients of the education variables, thus there was little evidence of a mediating effect of these factors. Among Black girls, we were unable to examine the mediating effect of alcohol use during pregnancy due to small cells (i.e. only four girls ever used alcohol during pregnancy). Among non-Black girls, the addition of alcohol use during pregnancy did not change the regression coefficients of the education variables (See Additional file 1: Table S1). There was no relationship between education variables and potential mediator variables. It would be interesting to explore alternative pathways that might explain the effect of adolescent educational aspiration and grade skipping on birth outcomes.

Surprisingly, we found that smoking during pregnancy was positively related to Black adolescent mothers’ birthweight and gestational age. This finding is inconsistent with extensive literature documenting increased risk of adverse birth outcomes with smoking during pregnancy [51]. It is possible that this unexpected result is due to the very small number of Black adolescent mothers who reported any smoking during pregnancy (n = 14), which may lead to spurious results. It may also be due to sample selection biases, since our sample was limited to mothers who experienced a pregnancy as an adolescent and had a singleton live birth, or reporting biases. Given these potential issues with the ascertainment of smoking during pregnancy, results should be interpreted with caution.

In the post-hoc analysis, we found that lower GPA was related with a higher risk of teen pregnancy in both Black and non-Black girls, and that higher educational aspiration was related with a lower risk of teen pregnancy in non-Black girls. Our findings were consistent with existing literature which suggests that better grades in school and/or higher educational aspirations are associated with postponed sexual activities [52-55]. On the other hand, Tucker et al. did not find a significant association between parental educational expectations and teen pregnancy using data from Add Health [56]. However, the authors used parents’ educational expectations [56] while we examined adolescents’ own educational aspiration. Among non-Black girls, those who ever repeated a grade were more likely to experience teen pregnancy. This is consistent with evidence from existing studies that students who are over-age for grade experience a higher level of emotional stress and more behavioral problems, including substance abuse and early sexual debut [57-59]. We found that grade skipping was related with a higher risk of having teen pregnancy among non-Black girls, which is an unexpected finding. There were 24 non-Black girls who ever skipped a grade and had teen pregnancy. With this relatively small cell size, the possibility that this finding is due to chance cannot be ruled out. These results suggest that adolescent girls’ academic performance and educational aspiration influence both the likelihood of getting pregnant as a teenager and birth outcomes of teenage pregnancy.

Our findings suggest that interventions that affect adolescents’ educational aspirations and grade acceleration might have positive effects on birth outcomes should the adolescent become pregnant. It has been found that both personal and social attributes are important factors that influence educational aspiration and grade acceleration in adolescent students [47,60,61]. Adolescents’ liking and enjoyment of school as well as a sense of affinity increases the probability of putting further education into the plan. A friendly atmosphere at school and perceived support from parents are also influential with regard to adolescents’ educational motivation [61]. Wells et al. [50] found that children with parents who more often discuss school programs, school activities, and things studied in class were more likely to have grade acceleration. Thus, the development of a supportive family and school environment is important in stimulating educational aspiration and promoting academic performance, which also has the benefit of being positively associated with birth outcomes among adolescent mothers. In addition, efforts should be made to identify and place students in appropriate learning environment/grades [27,50].

Our study used a large, nationally-representative dataset. Measurement of exposures was made prior to outcome data, which allows for the temporal inference between academic performance and educational expectation and the birth outcomes. Despite these strengths, our study has several limitations. First, we used adolescents’ self-reported letter grades rather than school transcript, which would have provided a more accurate and reliable measure of adolescents’ academic performance. The measurement of grade retention was also subject to reporting bias. Second, we reduced our sample size by requiring complete covariate data, which could bias our study results. Analyses comparing included (n = 763) versus excluded (n = 215) adolescents indicated no significant difference in birthweight (p = 0.71), gestational age (p = 0.62), race (p = 0.10), age at pregnancy (p = 0.16), parental education (p = 0.10), GPA (p = 0.41), grade skipping (p = 0.20), adolescents’ educational aspiration (p = 0.15), smoking during pregnancy (p = 0.47), and prenatal care visit (p = 0.17). However, there were significant differences in baseline age (15.33 ± 0.14 vs. 15.91 ± 0.20 in included and excluded sample, respectively; p < 0.001), baseline BMI (22.07 ± 0.19 vs. 22.94 ± 0.44 in included and excluded sample, respectively; p = 0.03), and percent of grade repeating (20.18% vs. 38.35% in included and excluded sample, respectively; p < 0.001). Third, we relied on maternal self-reports of both birthweight and gestational age, which may be subject to recall biases. However, past studies comparing birth records to maternal self-report have found maternal reports of birth outcomes are largely a valid method of obtaining data [62].

## Conclusions

In summary, we found that grade skipping was associated with higher offspring’s birthweight among Black adolescents, and with higher birthweight and gestational age among non-Black adolescents. Higher educational aspiration was associated with higher birthweight among non-Black adolescents. Future research that explores the pathways by which educational aspiration and grade skipping influence birth outcomes among adolescent mothers could add to our understanding of the determinants of outcomes of teen births. As is reflected in the new Healthy People 2020 goals related to adolescent success in school, a partnership between adolescent-serving clinicians, public health entities, and educational institutions is warranted to improve the well-being of both pregnant and non-pregnant adolescents.

## Abbreviations

GPA: Grade point average.

## Competing interests

The authors declare that they have no competing interests.

## Authors’ contributions

EWH and ASM developed the conception for the research. YX analyzed and interpreted the data and prepared the manuscript. EWH and ASM were involved in drafting and critically revising the manuscript. All authors read and approved the final version.

## Pre-publication history

The pre-publication history for this paper can be accessed here:

http://www.biomedcentral.com/1471-2393/14/3/prepub

## Supplementary Material

Additional file 1: Table S1 This file includes results of multivariable analysis between maternal characteristics, educational variables and birth outcomes among non-Black girls (n = 519) after the inclusion of three potential mediators: smoking during pregnancy, initiation of prenatal care, and alcohol use during pregnancy. Click here for file
