# Supplementary material for: Academic performance, educational aspiration and birth outcomes among adolescent mothers: a national longitudinal study
Source: BMC Pregnancy Childbirth. 2014 Jan 15;14:3. doi: 10.1186/1471-2393-14-3 (PMC3897950; doi:10.1186/1471-2393-14-3)
Supplement: Additional file 1: Table S1 — This file includes results of multivariable analysis between maternal characteristics, educational variables and birth outcomes among non-Black girls (n = 519) after the inclusion of three potential mediators: smoking during pregnancy, initiation of prenatal care, and alcohol use during pregnancy. [file 1471-2393-14-3-S1.docx]

| **Additional file 1: Table S1. Multivariable analysis between maternal characteristics, educational variables and birth outcomes among non-Black girls (N=519)** ^†^ | | | | | |
| --- | --- | --- | --- | --- | --- |
|  | Birthweight (kg) | |  | Gestational age (week) | |
| Baseline age |  | 0.001(0.02) |  |  | 0.004(0.10) |
| Age at pregnancy |  | -0.02(0.03) |  |  | -0.09(0.10) |
| Baseline BMI category |  |  |  |  |  |
| Underweight |  | -0.16(0.07) |  |  | -0.09(0.30) |
| Normal weight |  | ref |  |  | ref |
| Over weight |  | -0.11(0.13) |  |  | -0.08(0.47) |
| Obese |  | 0.06(0.11) |  |  | 0.65(0.37) |
| Parental education |  |  |  |  |  |
| ≥HS |  | ref |  |  | ref |
| less than HS |  | -0.07(0.10) |  |  | -0.40(0.28) |
| GPA |  |  |  |  |  |
| Below average |  | 0.12(0.07) |  |  | 0.35(0.30) |
| Above average |  | ref |  |  | ref |
| Ever skipped a grade |  |  |  |  |  |
| No |  | ref |  |  | ref |
| Yes |  | 0.49(0.17)** |  |  | 1.11(0.43)* |
| Ever repeated a grade |  |  |  |  |  |
| No |  | ref |  |  | ref |
| Yes |  | -0.07(0.08) |  |  | -0.30(0.35) |
| Educational aspiration |  | 0.08(0.04)* |  |  | 0.14(0.15) |
| Smoking during pregnancy |  |  |  |  |  |
| No |  | ref |  |  | ref |
| Yes |  | -0.05(0.07) |  |  | 0.10(0.23) |
| Initiation of prenatal care |  |  |  |  |  |
| 1^st^ trimester |  | ref |  |  | ref |
| 2^nd^ trimester |  | 0.06(0.06) |  |  | 0.54(0.25) |
| 3^rd^ trimester or no prenatal care |  | -0.02(0.12) |  |  | 0.22(0.43) |
| Alcohol use during pregnancy |  |  |  |  |  |
| No |  | ref |  |  | ref |
| Yes |  | 0.13(0.11) |  |  | 0.67(0.50) |
| *P<0.05; **P<0.01;  ^†^ Figures presented are regression coefficients with standard errors | | | | | |
